# Paternally Expressed, Imprinted Insulin-Like Growth Factor-2 in Chorionic Villi Correlates Significantly with Birth Weight

**DOI:** 10.1371/journal.pone.0085454

**Published:** 2014-01-15

**Authors:** Charalambos Demetriou, Sayeda Abu-Amero, Anna C. Thomas, Miho Ishida, Reena Aggarwal, Lara Al-Olabi, Lydia J. Leon, Jaime L. Stafford, Argyro Syngelaki, Donald Peebles, Kypros H. Nicolaides, Lesley Regan, Philip Stanier, Gudrun E. Moore

**Affiliations:** 1 Fetal Development and Growth Research Group, Clinical and Molecular Genetics Unit, Institute of Child Health, University College London, London, United Kingdom; 2 Department of Obstetrics and Gynaecology, St. Mary's Campus, Imperial College London, London, United Kingdom; 3 Institute for Women's Health, University College London, London, United Kingdom; 4 Harris Birthright Research Centre for Fetal Medicine, King's College Hospital, London, United Kingdom; VU University Medical Center, Netherlands

## Abstract

**Context:**

Fetal growth involves highly complex molecular pathways. IGF2 is a key paternally expressed growth hormone that is critical for *in utero* growth in mice. Its role in human fetal growth has remained ambiguous, as it has only been studied in term tissues. Conversely the maternally expressed growth suppressor, *PHLDA*2, has a significant negative correlation between its term placental expression and birth weight.

**Objective:**

The aim of this study is to address the role in early gestation of expression of *IGF1*, *IGF2*, their receptors *IGF1R* and *IGF2R*, and *PHLDA*2 on term birth weight.

**Design:**

Real-time quantitative PCR was used to investigate mRNA expression of *IGF1*, *IGF2*, *IGF1R*, *IGF2R* and *PHLDA2* in chorionic villus samples (CVS) (n = 260) collected at 11–13 weeks' gestation. Expression was correlated with term birth weight using statistical package R including correction for several confounding factors.

**Results:**

Transcript levels of *IGF2* and *IGF2R* revealed a significant positive correlation with birth weight (0.009 and 0.04, respectively). No effect was observed for *IGF1*, *IGF1R* or *PHLDA2* and birth weight. Critically, small for gestational age (SGA) neonates had significantly lower *IGF2* levels than appropriate for gestational age neonates (p = 3·6×10^−7^).

**Interpretation:**

Our findings show that *IGF2* mRNA levels at 12 weeks gestation could provide a useful predictor of future fetal growth to term, potentially predicting SGA babies. SGA babies are known to be at a higher risk for type 2 diabetes. This research reveals an imprinted, parentally driven rheostat for *in utero* growth.

## Introduction

Fetal growth involves a complex interaction between genes and the environment, with such significant complexity that many of the molecular pathways remain to be elucidated. Normal fetal growth depends on the successful nutrient exchange between the mother and the fetus via the placenta. When this critical balance is impaired it can result in a small for gestational age (SGA) baby [1]. SGA neonates have a reported incidence of ∼6% of pregnancies in developed countries and as high as 40% in some developing countries [2], [3]. SGA is also associated with an increased risk of neonatal death. Although the survivors do exhibit catch up growth, it is important to note that they remain at a higher risk for common, late onset chronic diseases such as type 2 diabetes (T2D) and cardiovascular disease [4]. Hales et al., (1991) reported a graded inverse association between birth weight and T2D risk with the highest risks of T2D occurring at the lowest levels of birth weight [5]. In a more recent meta-analysis, an increased risk for T2D (OR = 1.47) is seen for SGA versus appropriate for gestational age (AGA) neonates [6].

One group of growth genes of particular interest are the imprinted genes that are found almost exclusively in Eutherian (placental) mammals. Genomic imprinting defines allele-specific, differential expression of a gene, according to its parent of origin. If the paternal allele is expressed, the maternal allele is imprinted (silenced) and vice versa. The “parental conflict hypothesis” is still the most widely accepted explanation for the evolution of genomic imprinting [7]. It has been suggested that expression of the father's genes enhance fetal growth improving the success of the paternal genome to be passed on. In contrast, the mother's genome limits fetal growth, distributing equal resources to each of her offspring, whilst ensuring her own survival post birth allowing her to reproduce again. Studies of imprinted genes generally support this model, with one of the most striking examples being the reciprocal imprinting effects and associated growth patterns for mouse insulin-like growth factor 2 (*Igf2*) and its chelating receptor *Igf2r* [8]. In transgenic mice, loss of function of the paternally-expressed *Igf2* results in a 40% reduction in birth weight which contrasts with loss of the maternally-expressed *Igf2r*, resulting in a 30% increase in birth weight [9], [10]. In addition, *IGF2* has been implicated in two imprinted human growth disorders, the overgrowth, Beckwith-Wiedemann syndrome (BWS) [11] and the growth restricting Silver-Russell syndrome (SRS) [12].

Pleckstrin homology-like domain family A member 2 (*PHLDA2*) is maternally expressed, paternally imprinted, in both humans and mice [13]. Involvement of *Phlda2* in growth and development of the placenta was demonstrated by knockout mouse models that were associated with a significant increase in placental size during mid to late gestation [14]. Studies that have investigated the human placental expression of *PHLDA2* report increased expression in SGA pregnancies and a negative association with birth weight [15], [16], [17].

Insulin-like growth factor 1 (*IGF1*) and its primary binding receptor *IGF1R* are not imprinted. *IGF1* exerts its growth properties on almost every cell in the body. A homozygous partial deletion of *Igf1r* in mice stunted height and weight, as well as disrupted the pubertal growth spurt. A complete inactivation of *Igf1r* is lethal in the neonatal period [18]. In contrast to *Igf2* deficient mice, restriction of growth was seen to continue into the postnatal period in the *Igf1* mutants [19].

The analyses of human placental expression of *IGF1*, *IGF2*, *IG1R* and *IGF2R* have previously been confined to samples obtained at the time of birth. *IGF1* under-expression was observed in term placental samples from SGA pregnancies [20], while mutations in the *IGF1R* gene led to abnormalities in the function of IGF1 receptors that may also slow down intrauterine and subsequent growth in humans [21].

Studies on *IGF2* have reported conflicting results. Some demonstrate no correlation between *IGF2* expression and birth weight [16] whereas others have variably shown that in SGA pregnancies compared to controls, *IGF2* expression is either increased [22], decreased [15], [23], [24], or similar [25], including at the protein level [26]. Moreover, studies have either shown no significant relationship between IGF2 cord serum levels and size at birth [27], or a positive effect on birth weight [28]. In others, IGF2 cord blood levels were significantly correlated with birth weight only when its interaction with IGF2R was taken into account [29].

The profound role of the IGF1 and IGF2 pathways in the regulation of fetal growth have been established largely based on experiments using the mouse as a model [9], [10], [18]. Analysis in humans has been hampered by the lack of available tissue to study during the course of pregnancy and has therefore relied on the use of term placenta, at a time when this tissue has become redundant and therefore the levels of gene expression may no longer reliably reflect the needs of the growing baby. To fully assess the role of these growth factors and their receptors, we have focused on an earlier developmental time point when these genes are much more likely to measure functionally relevant expression levels.

## Methods

### Study population

Chorionic villus sampling (CVS) was performed at 11–13 weeks of gestation in 260 singleton pregnancies that subsequently resulted in normal live births at term. The samples were collected from women undergoing CVS for prenatal diagnosis of chromosomal defects at King's College Hospital London. The excess tissue samples used for this study were obtained from women agreeing to participate in research, which was approved by the King's College Hospital Ethics Committee.

Demographic characteristics were recorded including maternal age, racial origin, smoking status, parity and body mass index as well as pregnancy outcomes such as gestational age at delivery, sex and birth weight. Other birth parameters such as placental weight were not available, nor were blood or tissue samples from the newborn baby. The pregnancies were subdivided according to the birth weight of neonates into small for gestational age (SGA) with birth weight <10^th^ percentile, large (LGA) with birth weight >90^th^ percentile and appropriate (AGA).

### Preparation of DNA and RNA from chorionic villus samples

DNA and RNA were extracted using the iPrep Purification Instrument (Invitrogen), either by use of the iPrep™ ChargeSwitch® gDNA Tissue Kit, or iPrep™ PureLink™ Total RNA and Trizol® Plus RNA kit including DNAse treatment, according to the manufacturer's instructions.

### Reverse transcription

Reverse transcriptase (RT) methodology was based on a standard protocol using M-MLV reverse transcriptase and random primer hexamers (Promega). Primers for the housekeeping gene *β-actin* (*ACTB*) were used to check the integrity of the cDNA and ensure no DNA contamination. The forward (F) and reverse (R) primers spanned an intron and the sequences are: *β*-actin.F- gtcttcccctccatcgtg and *β*-actin.R- ggtcatcttctcgcggttg.

### Polymerase Chain Reaction (PCR)

Genomic DNA and cDNA from CVS were amplified by PCR before sequencing. Primers (5′ to 3′ sequence) used for genomic DNA are IGF2.F- aacaccccacaaaagctcag; IGF2.R- tgcatggattttggttttca; IGF2R.F- gaaacacaaaacctacgacc; IGF2R.R- agaacccaaaagagccaacc; PHLDA2.F- caaaccccgcacgccatgag and PHLDA2.R- ctgtgcccattgcaaataaatc. The same primers were used for cDNA with the exception of IGF2R.R- cctttggagtacgtgacaac. 20 ul reactions were set up and thermal cycling conditions were 94°C for 5 min, 94°C for 30 sec, 60°C for 30 sec, 72°C for 30 sec for 35 cycles, and 72°C for 2 min.

### Imprinting analysis

Imprinting analysis of *IGF2*, *IGF2R* and *PHLDA2* in CVS was carried out by DNA sequence analysis of expressed single nucleotide polymorphisms (SNP) in CVS gDNA after amplification using specific primers (IGF2.F- aacaccccacaaaagctcag; IGF2R.R- cctttggagtacgtgacaac and PHLDA2.F- caaaccccgcacgccatgag). Corresponding cDNA samples were screened in patients heterozygous for the *IGF2* A/G (rs680), *IGF2R* A/G (rs1805075) and *PHLDA2* A/G (rs1056819) SNPs (http://www.ncbi.nlm.nih.gov/projects /SNP/). Sequencing reactions were prepared according to the manufacturer's instructions (Applied Biosystems) using the ABI Prism Big Dye terminator cycle sequencing ready reaction kit (BDT v1.1). Sequencing products were run on an ABI Prism 3730 DNA analyzer, and the read-out was analysed with Sequencher™ v4.8 (Gene Codes Corporation).

### Real-time quantitative PCR

The quantitative expression analysis of the genes of interest, *IGF1*, *IGF2*, *IGF1R*, *IGF2R* and *PHLDA2* as well as the endogenous control gene *L19* (a housekeeping gene ubiquitously expressed in the placenta) [16] was determined by real-time quantitative PCR (RTqPCR) with SYBR Green (ABI) using the StepOnePlus Real-Time PCR System (ABI). Primers (5′ to 3′ sequence) used for quantitative analysis are IGF1.F- ggaggctggagatgtattgc; IGF1.R- acttgcttctgtcccctcct; IGF2.F- cgagagggacgtgtcgacc; IGF2.R- ggactgcttccaggtgtcata; IGF2R.F- ccggcgtgctctgga; IGF2R.R- ccagagggtcacagtggaaga; IGF1R.F- ccaagggtgtggtgaaagat; IGF1R.R- tccatgatgaccagtgttgg; PHLDA2.F- ccatccccgcagcccaaacc; PHLDA2.R- ccacgtcctagcctgggtcc; L19.F- gcggaagggtacagccaat and L19.R- caggctgtgatacatgtggcg. All primer sets were free of primer-dimer products. The RTqPCR assays were run in triplicate for each sample, with the gene of interest and housekeeping gene run on the same 96-well plates. A control pool of CVS cDNA was also included on each plate. Amplification conditions include initial incubation at 95°C for 10 min and repetitive denaturation at 95°C for 15 sec and annealing at 60°C for 1 min for 40 cycles.

After amplification, quantitative expression levels were obtained using the StepOne software (version 2.1). All triplicate cycle threshold (C_T_) values were within 1 C_T_ of each other. The quantitative values for each triplicate were averaged and the relative expression of the genes of interest was determined by a ratio of their expression to that of the *L19* housekeeping gene for the same sample.

### Statistical analysis

Standard multiple linear regression models were used to examine the correlation of *IGF1*, *IGF2*, *IGF2R*, *IGF1R* and *PHLDA2* relative expression values to birth weight after adjustment for gestational age at delivery, sex, parity, maternal age, smoking status and maternal body mass index. Any outliers more than 2 standard deviations from the mean were removed. The analysis of variance (one-way ANOVA) was used to compare the gene expression means between the three different categorical birth weight groups (SGA, AGA, LGA) taking into consideration any confounding factors. Statistical package “R” (version 2.13.0) was used for the analyses and for calculating adjusted R-squared (R^2^) values. Significance was defined as p<0.05.

## Results

In this study, we have investigated the relationship between *IGF1*, *IGF2*, *IGF2R*, *IGF1R* and *PHLDA2* mRNA levels in first-trimester placental tissue with the birth weight of the resultant term new born babies. We correlate this data taking into account the carefully recorded birth parameters that may have a confounding effect (gestational age, sex, parity, maternal BMI and smoking status; figure S1). Regression analysis was performed after standardization to the endogenous control gene *L19*, in relation to birth weight corrected for confounding factors.

**Figure 1:**
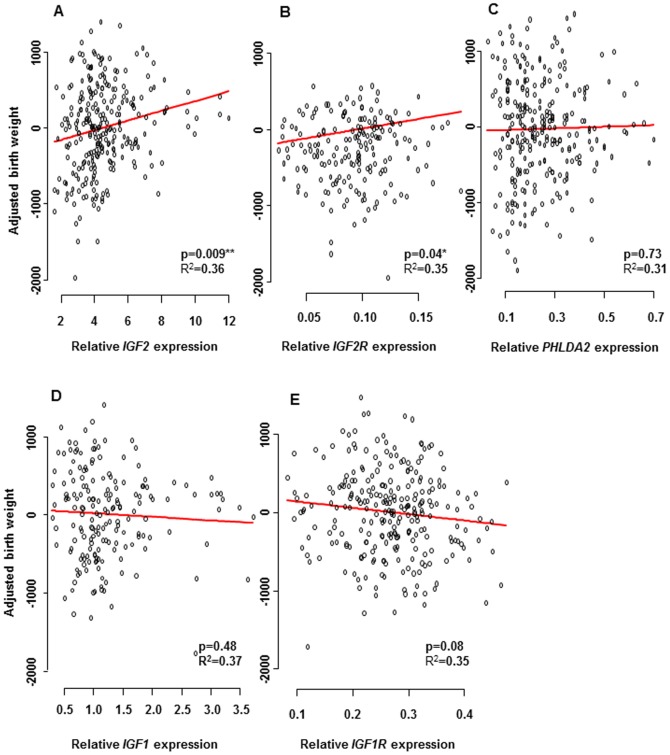
mRNA expression levels of *IGF2*, *IGF2R*, *PHLDA2*, *IGF1* and *IGF1R* in chorionic villi. The expression levels of *IGF2*, *IGF2R*, *PHLDA2*, *IGF1* and *IGF1R* after standardization to the endogenous control gene *L19*, in relation to birth weight corrected for parity, sex, GA at term, maternal BMI and smoking status. Significant associations were observed for CVS expression of A) *IGF2* (p = 0.009) and B) *IGF2R* (p = 0.04). No significant association was found for C) *PHLDA2* (p = 0.73), for D) *IGF1* (p = 0.48) or for E) *IGF1R* (p = 0.08).

Significant associations were observed for expression of *IGF2* (p = 0.009) and *IGF2R* (p = 0.04) between CVS tissue and birth weight (figure 1A–B). Importantly the range of *IGF2* in relative expression units was from 1.5–12 with one relative expression unit being equivalent to a change in birth weight of 63 g. No association was found for *PHLDA2* (p = 0.73), *IGF1* (p = 0.48) or *IGF1R* (p = 0.08) between CVS tissue and birth weight (figure 1C–E).

**Figure 2:**
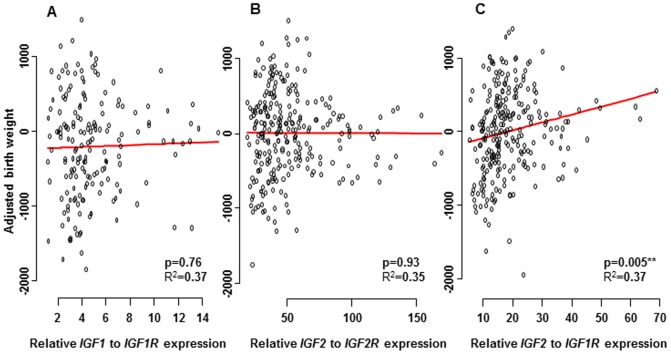
mRNA expression levels of *IGF1/IGF1R*, *IGF2*/*IGF2R* and *IGF2/IGF1R* in chorionic villi. The expression levels of *IGF1/IGF1R*, *IGF2/IGF2R* and *IGF2/IGF1R* after standardization to the endogenous control gene *L19*, in relation to birth weight corrected for parity, sex, GA at term, maternal BMI and smoking status. No significant association was found for A) the ratio of *IGF1* to *IGF1R* (p = 0.76), or for B) the ratio of *IGF2* to *IGF2R* (p = 0.93). Significant association was observed for CVS expression of C) the ratio of *IGF2* to *IGF1R* (p = 0.005) and birth weight.

As both IGF1 and IGF2 bind to the IGF1R and only IGF2 binds to the IGF2R we decided to look at this relationship between the ligands and their receptors and any correlation they might have with birth weight. The ratio of *IGF2 to IGF1R* (p = 0.005) was significantly associated with birth weight but no association was found for the ratio of *IGF1 to IGF1R* (p = 0.76) or the ratio of *IGF2 to IGF2R* (p = 0.93) between CVS tissue and birth weight (figure 2).

**Figure 3:**
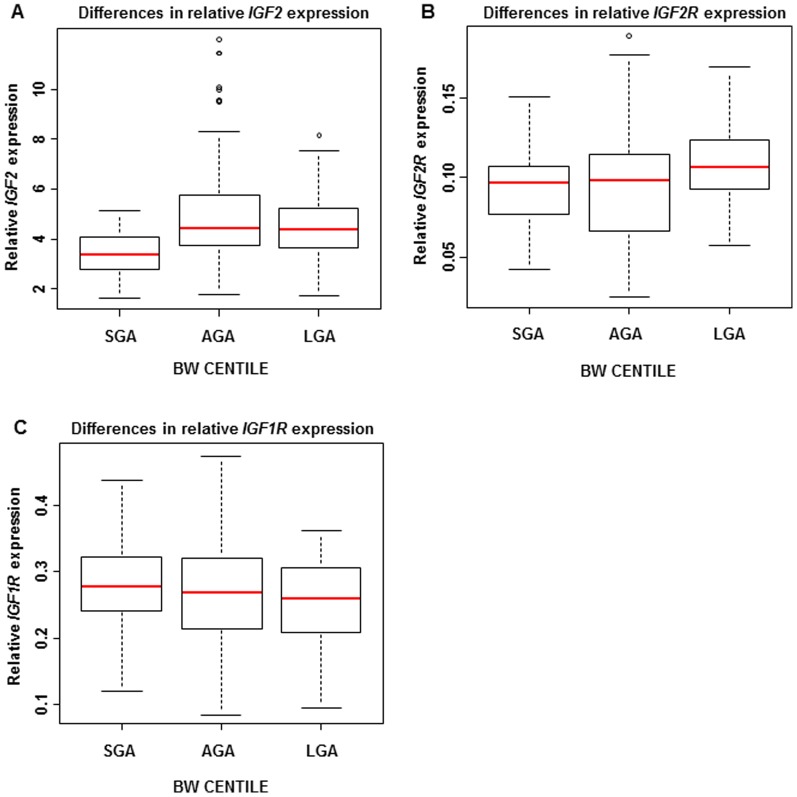
mRNA expression levels of *IGF2*, *IGF2R*, and *IGF1R* in chorionic villi according to BW centile. Relative *IGF2*, *IGF2R*, and *IGF1R* expression levels in the pregnancies with small (SGA), appropriate (AGA) and large (LGA) neonates. In the SGA group, compared to the AGA group, A) *IGF2* expression was lower (p = 3·6×10^−7^), but there was no significant difference in the expression levels of B) *IGF2R* (p = 0·76) or C) *IGF1R* (p = 0·15). In the LGA group B) *IGF2R* expression was higher than in the AGA group (p = 0·02) and C) *IGF1R* expression was lower than in the SGA group (p = 0·05).

To investigate the whole growth spectrum we subdivided the pregnancies into small for gestational age (SGA; n = 50) with birth weight <10^th^ percentile, large (LGA; n = 65) with birth weight >90^th^ percentile and appropriate (AGA; n = 145). In the SGA group compared to the AGA group, *IGF2* expression was reduced by statistically significant amounts (p = 3.6×10^−7^), despite no significant differences in the expression levels of individual receptors (*IGF2R*, p = 0.76 or *IGF1R*, p = 0.15). In the LGA group *IGF2R* expression was found to be higher than in the AGA group (p = 0.02) and *IGF1R* expression was lower than in the SGA group (p = 0.05) (figure 3).

**Figure 4:**
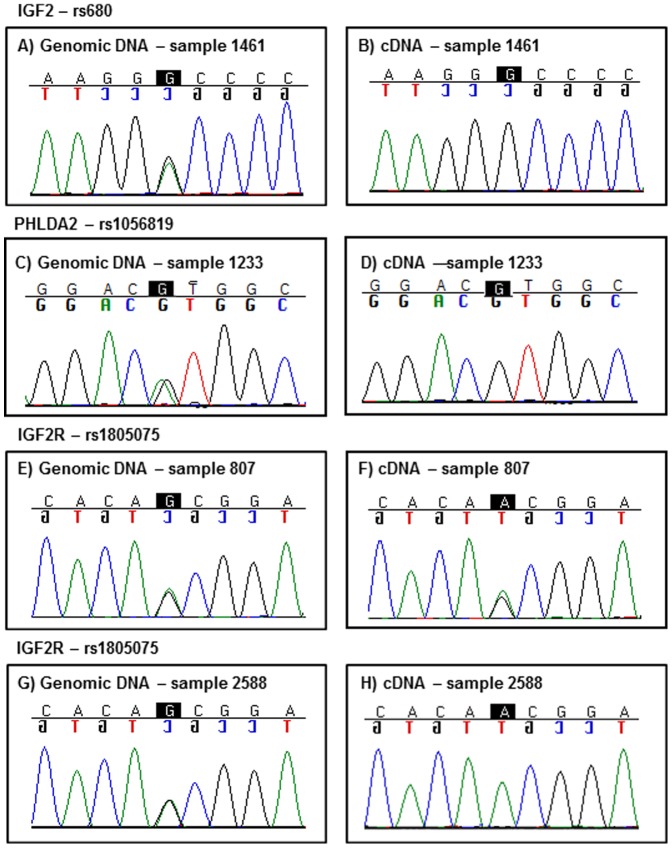
Imprinting analysis of *IGF2*, *PHLDA2* and *IGF2R*. Representative sequencing chromatograms are shown from one of the 40 informative CVS samples for the *IGF2* A/G polymorphism (rs680) (A, B), one (from 21) for the *PHLDA2* A/G polymorphism (rs1056819) (C, D) and two (from 24) for the *IGF2R* A/G polymorphism (rs1805075) (E, F, G, H). Heterozygous genomic CVS DNA sequence is shown in A), C), E) and G). While monoallelic expression from the corresponding cDNA samples are shown in B), D) and H). Monoallelic expression in cDNA was found in all informative *IGF2* and *PHLDA2* samples as well as 12% of *IGF2R* samples. Biallelic expression, as shown in F), was found in the majority (88%) of informative cDNA samples for *IGF2R*.

Given that *IGF2* and *PHLDA2* are both known to be imprinted in mouse and human [8], [13], [30], we wanted to confirm that monoallelic expression was maintained in CVS material, since reversion to biallelic expression may impact on mRNA levels. Furthermore, *IGF2R* in the human population is reported to be monoallelically expressed (i.e. imprinted) in only 10% of individuals, which is in contrast to the mouse, where it is fully imprinted [31]. We therefore wished to confirm its imprinting status in CVS material and to investigate for any potential skewing. The transcribed SNPs rs680 (*IGF2*), rs1805075 (*IGF2R*) and rs1056819 (*PHLDA2*) were used to report on allele-specific expression and thus determine imprinting status of *IGF2*, *IGF2R* and *PHLDA2*. Genomic DNA extracted from the CVS from 200 patients were tested for heterozygosity at these SNPs, with 40 samples found to be informative at rs680 for *IGF2*, 24 samples at rs1805075 for *IGF2R* and 21 samples at rs1056819 for *PHLDA2*. The 40 informative *IGF2* individuals and the 21 informative *PHLDA2* individuals were all found to be monoallelically expressed. For the 24 samples informative for *IGF2R*, 21 (88%) were biallellically expressed for *IGF2R* and 3 (12%) were monoallelic, which is similar to the expected population frequency (figure 4). Thus, skewed monoallelic/biallelic expression was unlikely to be a factor reflected in the relative expression levels associated with birth weight.

## Discussion

Previous studies have linked *IGF2* expression to birth weight, by showing that *IGF2* in term placenta is decreased in SGA pregnancies compared to controls [23], [24]. However, the first trimester *IGF2* expression data reported in this study, provides the first evidence for the role of this paternally expressed imprinted gene as an *in utero* fetal growth enhancer this early in human pregnancy. This is compatible with results previously described in animal studies [9], [32]. For example, in mouse experiments, paternally expressed *Igf2* was reported to control 40% of fetal growth, with the maternally expressed gene *Igf2r* limiting fetal growth [10], [33]. In humans, *IGF2* is consistently maternally imprinted and therefore a paternal-expression driven growth promoter. However, in humans, *IGF2R* is polymorphic for its imprinting status with 90% of individuals showing biallelic expression [31]. This indicates a possible shift in the mechanism of its regulation of expression away from that used in the mouse. Interestingly, here, the samples showing monallelic expression of *IGF2R* had similar levels to those showing biallelic expression.

As fetal size is subject to both positive and negative regulation, *IGF2* and *IGF2R* genes can exert opposite forces on the fetus, achieving a fine degree of control over the growth process. As levels of *IGF2* increase in the fetus, the levels of *IGF2R* can also increase, resulting in clearance of IGF2 from plasma and tissue fluids, correcting and maintaining levels of IGF2 in the circulation. This acts as a fine-tuning rheostat designed to prevent babies from overgrowth in order to maintain a normal growth trajectory. This is also supported by the fact that no association was observed between the *IGF2/IGF2R* expression levels and birth weight, as the ligand and the receptor work tightly together to regulate growth. The same applies for IGF1 and its regulating receptor IGF1R.

In this study, expression of *IGF1* in chorionic villi was not associated with birth weight outcome. These results directly contradict findings from studies involving mouse models which highlighted *IGF1* role in the regulation of both pre- and postnatal growth [34]. There also remains a strong possibility that *IGF1* is indeed relevant to intrauterine growth in the later stages of pregnancy as it is preparing the baby for a postnatal life. While fetal chorionic fetal samples in the present cohort proved an opportunity to assess correlation between gene expression and fetal growth in early gestation (11–13 weeks), this narrow window might not be the best to investigate *IGF1* expression, as IGF1 does not appear in the fetal circulation until later [35].

Although previous studies have shown that placental *PHLDA2* expression is negatively associated with birth weight [16], [17], in our study no statistically significant association was observed between *PHLDA2* expression levels in CVS and birth weight. This suggests that maternally expressed *PHLDA2* is suppressing the baby's growth later in pregnancy rather than early on. Previous studies investigating term placental *IGF2* expression have shown inconclusive results. However, in our study a statistically significant association was observed between *IGF2* expression levels in CVS and birth weight, suggesting that the paternal genome is promoting the baby's growth earlier in pregnancy. Therefore, the two parental genomes appear to be acting at different times during pregnancy to control the fetal weight. This supports the idea that whilst increased fetal growth is important early on, it must still require careful regulation by the mother to ensure a successful birth.

In our study, SGA neonates had significantly lower *IGF2* expression levels compared to AGA neonates, but no differences were observed between the levels of the receptors *IGF2R* and *IGF1R*. This highlights the importance of the *IGF2* ligand rather than the receptor level in determining size of the neonate in SGA pregnancies. This is in agreement with previous studies investigating *IGF2* mRNA levels in the placentas from growth-restricted pregnancies [23], [24].

To better understand the causes and consequences of fetal growth restriction as a human pregnancy complication, we focused on available tissue from the first trimester, as this time window is more likely to accurately reflect the *in utero* growth potential. We used a larger sample size and we investigated the whole growth spectrum including not only SGA but also LGA neonates. We reasoned that extending the investigation of these genes to samples displaying macrosomic birth weight would also reveal further correlations between growth parameters and gene expression. In this study, LGA neonates had higher *IGF2R* expression levels compared to AGA neonates and lower *IGF1R* levels compared to SGA babies. Similar results have been reported in placenta tissue for *IGF1R* mRNA levels alone [36], and this suggests that fetuses with high birth weight are producing more *IGF2R* and correspondingly less *IGF1R*, which could remove *IGF2*. This balance may represent another important compensatory mechanism in response to fetal overgrowth. This is also supported by the fact that a significant association was observed between the ratio of *IGF2* to *IGF1R* and birth weight, suggesting that the levels of IGF1R in LGA neonates decrease as their IGF2 levels increase, to avoid any further increase in size.

The “small baby syndrome hypothesis” proposed by Barker et al., (1993) suggests that there is an inverse linear relation between birth weight and T2D [37]. This is also supported by a large meta-analysis [38]. The mechanisms by which birth weight is related to T2D is still under debate, Barker et al. claim that this relationship reflects long-term consequences of under-nutrition *in utero* [37], whereas others do not see under-nutrition as playing a significant role [39]. Interestingly, specific fetal *IGF2* paternal haplotypes are linked to higher maternal glucose levels that increase the risk of gestational diabetes [40]. Nevertheless the amount of IGF2 available from the placenta early in the first trimester is likely to be a major factor in promoting growth.

An important aim for antenatal care is the prediction, detection and treatment of anomalous fetal growth. We know that variation in size at birth results from interaction between fetal genetic factors and the maternal genetic and uterine environment. In this study we also implicate the important role of the father's genes in fetal growth *in utero*. Understanding the developmental role, function and parent-of-origin effect of critical imprinted genes during human fetal growth will make an important contribution to effective clinical evaluation of growth disorders and their association with longer term, metabolically related, health risks such as T2D. Determination of paternal *IGF2* expression in the first trimester, potentially in maternal blood, may act as a predictor of fetal growth trajectory for later in the pregnancy. An early growth biomarker could be invaluable to alert Obstetricians and Neonatologists towards closer ‘at risk’ pregnancy surveillance.

## Supporting Information

Figure S1 **Confounding factors.** Correlation of birth weight with gestational age at term (p =  4.8×10^−15^; Fig. S1A), with maternal BMI (p = 0.0012; Fig. S1B), with gender (p = 1.4×10^−7^; Fig. S1C), with parity (p = 7.5×10^−5^; Fig. S1D), and maternal smoking status (p = 0.33; Fig S1E).
(TIF)

## References

[1] Pollack RN, Divon MY (1992) Intrauterine growth retardation: definition, classification, and etiology. Clin Obstet Gynecol 35: 99–107. 1544253 10.1097/00003081-199203000-00015

[2] Brodsky D, Christou H (2004) Current concepts in intrauterine growth restriction. J Intensive Care Med 19: 307–319. 15523117 10.1177/0885066604269663

[3] Albertsson-Wikland K, Wennergren G, Wennergren M, Vilbergsson G, Rosberg S (1993) Longitudinal follow-up of growth in children born small for gestational age. Acta Paediatr 82: 438–443. 8518519 10.1111/j.1651-2227.1993.tb12718.x

[4] Barker DJ (1992) Fetal growth and adult disease. Br J Obstet Gynaecol 99: 275–276. 1581269 10.1111/j.1471-0528.1992.tb13719.x

[5] Hales CN, Barker DJ, Clark PM, Cox LJ, Fall C, et al (1991) Fetal and infant growth and impaired glucose tolerance at age 64. BMJ 303: 1019–1022. 1954451 10.1136/bmj.303.6809.1019 PMC1671766

[6] Harder T, Rodekamp E, Schellong K, Dudenhausen JM, Plagemann A (2007) Birth weight and subsequent risk of type 2 diabetes: A meta-analysis. Am J Epidemiol 165: 849–857. 17215379 10.1093/aje/kwk071

[7] Moore T, Haig D (1991) Genomic imprinting in mammalian development: a parental tug-of-war. Trends Genet 7: 45–49. 2035190 10.1016/0168-9525(91)90230-N

[8] Willison K (1991) Opposite imprinting of the mouse Igf2 and Igf2r genes. Trends Genet 7: 107–109. 1648806 10.1016/0168-9525(91)90441-r

[9] DeChiara TM, Efstratiadis A, Robertson EJ (1990) A growth-deficiency phenotype in heterozygous mice carrying an insulin-like growth factor II gene disrupted by targeting. Nature 345: 78–80. 2330056 10.1038/345078a0

[10] Wang ZQ, Fung MR, Barlow DP, Wagner EF (1994) Regulation of embryonic growth and lysosomal targeting by the imprinted Igf2/Mpr gene. Nature 372: 464–467. 7984240 10.1038/372464a0

[11] Weksberg R, Shen DR, Fei YL, Song QL, Squire J (1993) Disruption of insulin-like growth factor-2 in Beckwith Wiedemann syndrome. Nat Genet 5: 143–149. 8252039 10.1038/ng1093-143

[12] Eggermann T, Meyer E, Obermann C, Heil I, Schuler H, et al (2005) Is maternal duplication of 11p15 associated with Silver-Russell syndrome? J Med Genet 42: e26. 15863658 10.1136/jmg.2004.028936 PMC1736048

[13] Qian N, Frank D, O'Keefe D, Dao D, Zhao L, et al (1997) The IPL gene on chromosome 11p15.5 is imprinted in humans and mice and is similar to TDAG51, implicated in Fas expression and apoptosis. Hum Mol Genet 6: 2021–2029. 9328465 10.1093/hmg/6.12.2021

[14] Frank D, Fortino W, Clark L, Musalo R, Wang W, et al (2002) Placental overgrowth in mice lacking the imprinted gene Ipl. Proc Natl Acad Sci USA 99: 7490–7495. 12032310 10.1073/pnas.122039999 PMC124258

[15] McMinn J, Wei M, Schupf N, Cusmai J, Johnson EB, et al (2006) Unbalanced placental expression of imprinted genes in human intrauterine growth restriction. Placenta 27: 540–549. 16125225 10.1016/j.placenta.2005.07.004

[16] Apostolidou S, Abu-Amero S, O'Donoghue K, Frost J, Olafsdottir O, et al (2007) Elevated placental expression of the imprinted PHLDA2 gene is associated with low birth weight. J Mol Med 85: 379–387. 17180344 10.1007/s00109-006-0131-8

[17] Ishida M, Monk D, Duncan AJ, Abu-Amero S, Chong J, et al (2012) Maternal inheritance of a promoter variant in the imprinted PHLDA2 gene significantly increases birth weight. Am J Hum Genet 90: 715–719. 22444668 10.1016/j.ajhg.2012.02.021 PMC3322226

[18] Klammt J, Pfaffle R, Werner H, Kiess W (2008) IGF signaling defects as causes of growth failure and IUGR. Trends Endocrinol Metab 19: 197–205. 18515143 10.1016/j.tem.2008.03.003

[19] Le Roith D, Scavo L, Butler A (2001) What is the role of circulating IGF-I? Trends Endocrinol Metab 12: 48–52. 11167121 10.1016/s1043-2760(00)00349-0

[20] Koutsaki M, Sifakis S, Zaravinos A, Koutroulakis D, Koukoura O, et al (2011) Decreased placental expression of hPGH, IGF-I and IGFBP-1 in pregnancies complicated by fetal growth restriction. Growth Horm IGF Res 21: 31–36. 21212012 10.1016/j.ghir.2010.12.002

[21] Abuzzahab MJ, Schneider A, Goddard A, Grigorescu F, Lautier C, et al (2003) IGF-I receptor mutations resulting in intrauterine and postnatal growth retardation. N Engl J Med 349: 2211–22. 14657428 10.1056/NEJMoa010107

[22] Abu-Amero SN, Ali Z, Bennett P, Vaughan JI, Moore GE (1998) Expression of the insulin-like growth factors and their receptors in term placentas: a comparison between normal and IUGR births. Mol Reprod Dev 49: 229–235. 9491374 10.1002/(SICI)1098-2795(199803)49:3<229::AID-MRD2>3.0.CO;2-Q

[23] Koukoura O, Sifakis S, Soufla G, Zaravinos A, Apostolidou S, et al (2011) Loss of imprinting and aberrant methylation of IGF2 in placentas from pregnancies complicated with fetal growth restriction. Int J Mol Med 28: 481–487. 21805044 10.3892/ijmm.2011.754

[24] Guo L, Choufani S, Ferreire J, Smith A, Chitayat D, et al (2008) Altered gene expression and methylation of the human chromosome 11 imprinted region in small for gestational age (SGA) placentae. Developmental Biology 320: 79–91. 18550048 10.1016/j.ydbio.2008.04.025

[25] Street ME, Seghini P, Fieni S, Ziveri MA, Volta C, et al (2006) Changes in interleukin-6 and IGF system and their relationships in placenta and cord blood in newborns with fetal growth restriction compared with controls. Eur J Endocrinol 155: 567–574. 16990656 10.1530/eje.1.02251

[26] Laviola L, Perrini S, Belsanti G, Natalicchio A, Montrone C, et al (2005) Intrauterine growth restriction in humans is associated with abnormalities in placental insulin-like growth factor signaling. Endocrinology 146: 1498–1505. 15564321 10.1210/en.2004-1332

[27] Klauwer D, Blum WF, Hanitsch S, Rascher W, Lee PD, et al (1997) IGF-I, IGF-II, free IGF-I and IGFBP-1, -2 and -3 levels in venous cord blood: relationship to birthweight, length and gestational age in healthy newborns. Acta Paediatr 86: 826–833. 9307161 10.1111/j.1651-2227.1997.tb08605.x

[28] Smerieri A, Petraroli M, Ziveri MA, Volta C, Bernasconi S, et al (2011) Effects of cord serum insulin, IGF-II, IGFBP-2, IL-6 and cortisol concentrations on human birth weight and length: pilot study. PLoS One 6: e29562. 22242132 10.1371/journal.pone.0029562 PMC3248435

[29] Ong K, Kratzsch J, Kiess W, Costello M, Scott C, et al (2000) Size at birth and cord blood levels of insulin, insulin-like growth factor I (IGF-I), IGF-II, IGF-binding protein-1 (IGFBP-1), IGFBP-3, and the soluble IGF-II/mannose-6-phosphate receptor in term human infants. The ALSPAC Study Team. Avon Longitudinal Study of Pregnancy and Childhood. J Clin Endocrinol Metab 85: 4266–4269. 11095465 10.1210/jcem.85.11.6998

[30] Giannoukakis N, Deal C, Paquette J, Goodyer CG, Polychronakos C (1993) Parental genomic imprinting of the human IGF2 gene. Nat Genet 4: 98–101. 8099843 10.1038/ng0593-98

[31] Monk D, Arnaud P, Apostolidou S, Hills FA, Kelsey G, et al (2006) Limited evolutionary conservation of imprinting in the human placenta. Proc Natl Acad Sci USA 103: 6623–6628. 16614068 10.1073/pnas.0511031103 PMC1564202

[32] Constância M, Hemberger M, Hughes J, Dean W, Ferguson-Smith A, et al (2002) Placental-specific IGF-II is a major modulator of placental and fetal growth. Nature 417: 945–948. 12087403 10.1038/nature00819

[33] Lau MM, Stewart CE, Liu Z, Bhatt H, Rotwein P, et al (1994) Loss of the imprinted IGF2/cation-independent mannose 6-phosphate receptor results in fetal overgrowth and perinatal lethality. Genes Dev 8: 2953–2963. 8001817 10.1101/gad.8.24.2953

[34] Le Bouc Y, Gircquel C, Holzenberger M (2003) Physiology of somatotropic axis: interest of gene inactivation experiments. Bull Acad Natl Med 187: 1225–43. 15146601

[35] Demendi C, Börzsönyi B, Nagy ZB, Rigó J Jr, Pajor A, et al (2011) Gene expression patterns of insulin-like growth factor 1, 2 (IGF-1, IGF-2) and insulin-like growth factor binding protein 3 (IGFBP-3) in human placenta from preterm deliveries: influence of additional factors. Eur J Obstet Gynecol 160: 40–44. 10.1016/j.ejogrb.2011.10.005 22071113

[36] Iniguez G, Gonzalez CA, Argandona F, Kakarieka E, Johnson MC, et al (2010) Expression and protein content of IGF-I and IGF-I receptor in placentas from small, adequate and large for gestational age newborns. Horm Res Paediatr 73: 320–327. 20389101 10.1159/000308163

[37] Barker DJ, Hales CN, Fall CH, Osmond C, Phipps K, et al (1993) Type 2 (non-insulin-dependent) diabetes mellitus, hypertension and hyperlipidaemia (syndrome X): relation to reduced fetal growth. Diabetologia 36: 62–67. 8436255 10.1007/BF00399095

[38] Whincup PH, Kaye SJ, Owen CG, Huxley R, Cook DG, et al (2008) Birth weight and risk of type 2 diabetes A systematic review. JAMA 300: 2886–2897. 19109117 10.1001/jama.2008.886

[39] Hofman PL, Regan F, Jackson WE, Jefferies C, Knight DB, et al (2004) Premature birth and later insulin resistance. N Engl J Med 351: 2179–2186. 15548778 10.1056/NEJMoa042275

[40] Petry CJ, Seear RV, Wingate DL, Manico L, Acerini CL, et al (2011) Associations between paternally transmitted fetal IGF2 variants and maternal circulating glucose concentartions in pregancy. Diabetes 60: 3090–3096. 21926269 10.2337/db11-0689 PMC3198064

