# Supplementary figures and images for: Paternally Expressed, Imprinted Insulin-Like Growth Factor-2 in Chorionic Villi Correlates Significantly with Birth Weight
Source: PLoS One. 2014 Jan 15;9(1):e85454. doi: 10.1371/journal.pone.0085454 (PMC3893199; doi:10.1371/journal.pone.0085454)

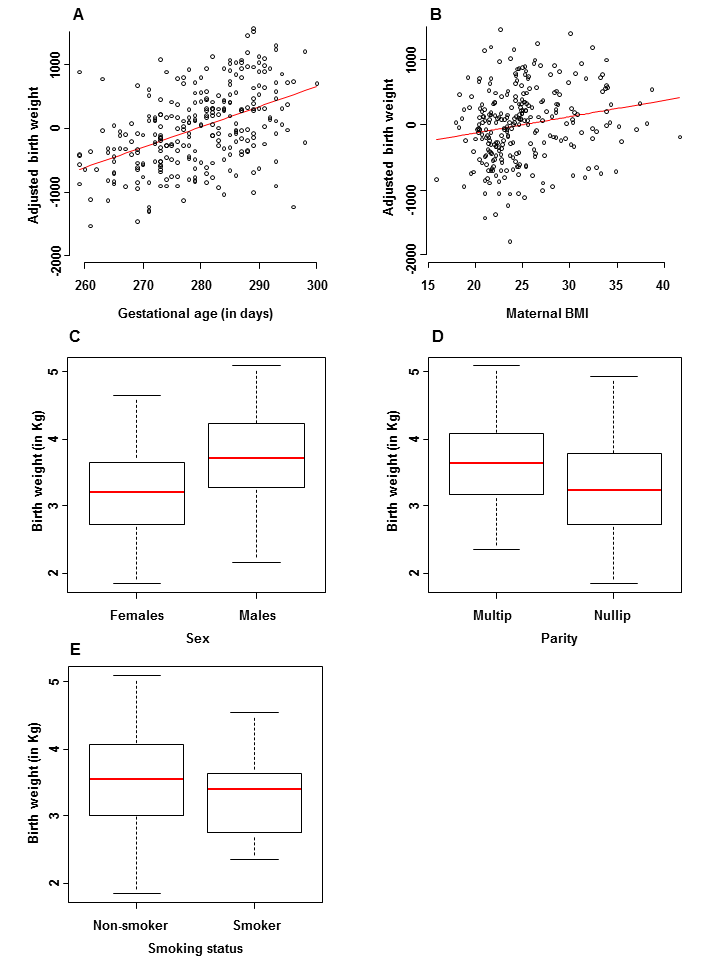

Supplement: Figure S1 — Confounding factors. Correlation of birth weight with gestational age at term (p = 4.8×10^−15^; Fig. S1A), with maternal BMI (p = 0.0012; Fig. S1B), with gender (p = 1.4×10^−7^; Fig. S1C), with parity (p = 7.5×10^−5^; Fig. S1D), and maternal smoking status (p = 0.33; Fig S1E). (TIF) [file pone.0085454.s001.tif]
